# Differences in Reach and Attrition Between Web-Based and Print-Delivered Tailored Interventions Among Adults over 50 Years of Age: Clustered Randomized Trial

**DOI:** 10.2196/jmir.2229

**Published:** 2012-12-17

**Authors:** Denise Astrid Peels, Catherine Bolman, Rianne Henrica Johanna Golsteijn, Hein De Vries, Aart Nicolaas Mudde, Maartje Marieke van Stralen, Lilian Lechner

**Affiliations:** ^1^Open University of The NetherlandsDepartment of PsychologyHeerlenNetherlands; ^2^Maastricht University Medical CentreDepartment of Health PromotionMaastricht UniversityMaastrichtNetherlands; ^3^Care and Public Health Research institute (Caphri)Maastricht UniversityMaastrichtNetherlands; ^4^EMGO Institute for Health and Care ResearchDepartment of Public and Occupational HealthVU University Medical CentreAmsterdamNetherlands

**Keywords:** Web-based, print-delivered, physical activity, older adults, response, reach, adoption, attrition, dropout, tailored advice

## Abstract

**Background:**

The Internet has the potential to provide large populations with individual health promotion advice at a relatively low cost. Despite the high rates of Internet access, actual reach by Web-based interventions is often disappointingly low, and differences in use between demographic subgroups are present. Furthermore, Web-based interventions often have to deal with high rates of attrition.

**Objective:**

This study aims to assess user characteristics related to participation and attrition when comparing Web-based and print-delivered tailored interventions containing similar content and thereby to provide recommendations in choosing the appropriate delivery mode for a particular target audience.

**Methods:**

We studied the distribution of a Web-based and a print-delivered version of the Active Plus intervention in a clustered randomized controlled trial (RCT). Participants were recruited via direct mailing within the participating Municipal Health Council regions and randomized to the printed or Web-based intervention by their region. Based on the answers given in a prior assessment, participants received tailored advice on 3 occasions: (1) within 2 weeks after the baseline, (2) 2 months after the baseline, and (3) within 4 months after the baseline (based on a second assessment at 3 months). The baseline (printed or Web-based) results were analyzed using ANOVA and chi-square tests to establish the differences in user characteristics between both intervention groups. We used logistic regression analyses to study the interaction between the user characteristics and the delivery mode in the prediction of dropout rate within the intervention period.

**Results:**

The printed intervention resulted in a higher participation rate (19%) than the Web-based intervention (12%). Participants of the Web-based intervention were significantly younger (*P*<.001), more often men (*P*=.01), had a higher body mass index (BMI) (*P*=.001) and a lower intention to be physically active (*P*=.03) than participants of the printed intervention. The dropout rate was significantly higher in the Web-based intervention group (53%) compared to the print-delivered intervention (39%, *P*<.001). A low intention to be physically active was a strong predictor for dropout within both delivery modes (*P*<.001). The difference in dropout rate between the Web-based and the printed intervention was not explained by user characteristics.

**Conclusions:**

The reach of the same tailored physical activity (PA) intervention in a printed or Web-based delivery mode differed between sociodemographic subgroups of participants over 50 years of age. Although the reach of the Web-based intervention is lower, Web-based interventions can be a good channel to reach high-risk populations (lower PA intention and higher BMI). While the dropout rate was significantly higher in the Web-based intervention group, no specific user characteristics explained the difference in dropout rates between the delivery modes. More research is needed to determine what caused the high rate of dropout in the Web-based intervention.

**Trial Registration:**

Dutch Trial Register (NTR): 2297: http://www.trialregister.nl/trialreg/admin/rctview.asp?TC=2297 (Archived by WebCite at http://www.webcitation.org/65TkwoESp).

## Introduction

During the last few decades, computer tailoring has become an important method to provide individuals with personalized health promotion advice [1]. Computer tailoring is a method that uses questionnaires to assess individual participants and to automatically produce feedback, based on the assessment, using computer-based data-driven decision rules. The feedback is automatically adapted to the personal characteristics of the participant [2,3]. Several studies confirmed the effectiveness of computer tailoring in terms of behavioral change by providing tailored health promotion advice [2,4,5].

While printed materials were previously one of the most used communication channels of tailored interventions, the Internet has become more popular in recent years. The Internet has the potential to provide large populations with individual advice at a relatively low cost and without intensive labor [6]. Recently, an enormous increase in Internet use has taken place in older age groups and lower socioeconomic status (SES) groups [7]. In Europe, Internet access ranges from 45% of the population in Bulgaria to 94% of the population in the Netherlands (where the current study was performed) [8]. Although home Internet access in the Netherlands is still relatively low (68%) for people over the age of 65 years, 91% of people between the age of 55 and 65 had home Internet access in 2010 [7]. Furthermore, differences in Internet access between SES groups have become much smaller (87% in low SES groups, 98% in high SES groups [7]). This indicates that low SES and old age are no longer barriers for Web-based interventions. Evidence-based Web-based interventions are particularly relevant for older age groups, as they are the fastest growing online user group and tend to have the most interest in and need for health-related subjects [9]. Of people over 55 years of age who use the Internet, 81% are interested in finding health information online [10].

Despite the high rates of Internet access, Web-based interventions do not often reach the intended target population. Different demographic groups, based on age or SES for example, use and respond to Web-based interventions differently [7,11,12]. The response rates to Web-based questionnaires are often significantly lower than the response rates to similar printed questionnaires [13-15]. Furthermore, Web-based interventions often experience high attrition rates. For Web-based interventions to achieve the optimal impact on public health, more insight related to reach and attrition of such interventions is of major importance. To our knowledge, no previous studies have been performed to compare the reach and attrition of a Web-based intervention to a print-delivered intervention targeted at a population over 50 years of age.

Several models have emphasized the importance of gaining insight into the reach and attrition of an intervention, including the RE-AIM framework [16,17] and McGuire’s persuasion-communication matrix [18]. The RE-AIM framework argues that the public impact of an intervention is a function of 5 factors: reach, efficacy, adoption, implementation, and maintenance [19]. According to McGuire’s persuasion-communication matrix [18], the channel of an intervention (ie, the delivery mode) and the characteristics of the user may influence decision making on the use of an intervention. Insight into the specific characteristics of the users of the intervention in different delivery modes could help future researchers choose the appropriate delivery mode for a particular target audience. It may also facilitate and optimize the adoption, reach, and impact of evidence-based tailoring of interventions for public health in the future.

To determine whether differences in reach and attrition rates are related to the delivery channel and not to the intervention itself, this study compares a Web-based and print-delivered tailored intervention with similar content. The Dutch Active Plus intervention, which can be delivered as a print or Web format, consists of tailored advice designed to stimulate physical activity (PA) among the aging population [20-23]. Previous studies have proven that the print-delivered intervention is effective in stimulating PA among people over the age of 50 [23,24]. The printed version was translated into a Web-based version [22], and the effectiveness of the online version is currently being evaluated. Preliminary results show that the printed and Web-based interventions are equally effective in promoting PA behavior after 6 months [25]. On 3 different occasions, the intervention provided tailored advice based on individual assessments from a questionnaire. Since this intervention contains multiple instances of advice, our study was concerned with both the reach of the intervention and the attrition to the follow-up advice. For thorough, overall insight into the impact of an intervention such as Active Plus, in addition to the effectiveness we need insight into the selective reach and attrition of the intervention in the different delivery modes [26].

Based on epidemiological evidence and previous studies, we expect that Web-based interventions will have a lower reach to the low SES and older age groups compared to print-delivered interventions [7,27-29]. It is not clear whether participation in Web-based interventions will be gender dependent in an older population. Earlier studies of tailored PA advice distributed to adults via the Internet showed that more women than men participated in Web-based interventions [12,30]. However, epidemiological evidence suggests that Internet access rates are higher among men in an older population [7,8]. The differing results from these studies may be due to differences in age, and more research will be needed to clarify the gender-dependent participation rates. This study provides insight into the differences in user characteristics related to the reach and attrition of a Web-based intervention compared to a print-delivered intervention among people over 50 years of age. Especially in older populations, Web-based tailored interventions might be more desirable and have advantages over printed tailored interventions. To our knowledge, no previous studies have investigated which user characteristics are related to the participation and attrition of a tailored intervention for an older population in different delivery modes. Differences in the usage and dropout rates between two delivery modes of an intervention can be a metric for the usability of a system and might be useful in determining the best distribution strategy [26]. Identifying which user characteristics are related to both the reach and attrition of an intervention can guide the appropriate selection of the delivery mode to a target subgroup, thereby increasing the public impact of future interventions.

## Methods

### Study Design

This study is part of a randomized controlled trial (RCT). For the assessment of the effectiveness of the intervention, a control group (which received no advice) was included in this study. Since the scope of this paper includes the reach and attrition of the intervention (which is compared between the Web-based and the print-delivered intervention group), the control group was excluded from this analysis.

**Figure 1:**
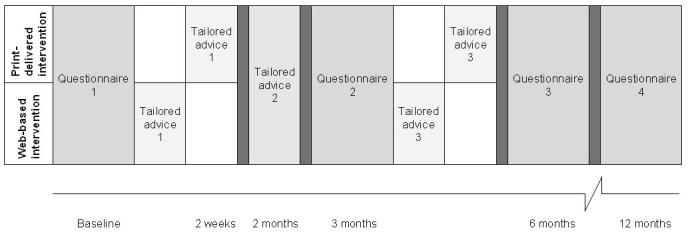
Timeline of the study.

Participants of both the print-delivered and Web-based intervention received tailored advice on 3 occasions, based on 2 questionnaires (written or Web-based) that they completed. Participants completed the first questionnaire at the start of the intervention (as input for the first and second sets of tailored advice) and a second questionnaire after 3 months (as input database for the third tailored advice). Figure 1 gives an overview of the timeline for this intervention. For analytical purposes, additional assessments took place at 6 and 12 months after the baseline, including a process evaluation (at the 6-month questionnaire) to assess how many individuals followed the advice. The 6- and 12-month questionnaires are not considered part of the intervention.

### Tailored Intervention

The Active Plus interventions are designed to stimulate or maintain PA among people over 50 years of age. These computer-tailored, theory- and evidence-based interventions were systematically developed using the intervention mapping protocol [20,22]. Originally, 2 print-delivered Active Plus interventions were developed and tested for effectiveness. The first was a basic computer-tailored intervention (tailored to personal and psychosocial characteristics). The second was the same intervention extended with additional environmental components that focused on giving tailored advice on local options and initiatives for being physically active. The additional environmental components intended to positively change peoples’ perceptions of the possibilities to be physically active in their own locale [21]. Both interventions aimed to influence awareness, initiation, and maintenance of PA by targeting premotivational constructs (ie, awareness and knowledge), motivational constructs (ie, attitude, self-efficacy, social influence, intrinsic motivation, and intention), and post-motivational constructs (ie, commitment, strategic planning, self-regulation skills, action planning, and coping planning) [20]. Previous studies showed that both print-delivered interventions were effective in stimulating PA among people over 50 years of age [23,24].

In 2010, both of the print-delivered interventions described above were adapted and translated into a Web-based intervention [22] using the RE-AIM framework. The content of the Web-based intervention was identical to that of the print-delivered interventions, but the display output of the interactive components in the Web-based version was different (eg, static modelling pictures were transferred into videos, a print-delivered map was transferred into Google Maps, and several hyperlinks were added).

Based on their answers given in the assessments [20,22], participants received tailored advice on 3 occasions (Figure 1): (1) within 2 weeks after the baseline assessment, (2) 2 months after the baseline assessment, and (3) within 4 months after the baseline assessment (based on the second assessment). The Web-based intervention participants received an email with a link that connected them with their online tailored advice. Additionally, they received an email with a copy of the personal advice in the same format as the print-delivered version. The tailored advice contained between 5 and 11 pages of text and illustrations, depending on changes in PA behavior and determinant scores. A more detailed description of the intervention content can be found elsewhere [20,22].

### Participants and Procedure

Intervention participants (adults over 50 years of age) were recruited via direct mailing in communities of the participating Municipal Health Council (MHC) regions (N=5; excluding control group participants). To prevent participants from the different intervention conditions contaminating each other, the intervention conditions were located in separate but comparable MHC regions. The regions were randomly assigned to one of the different intervention conditions (ie, Web-based basic, Web-based environment, printed basic, or printed environment). All participants were assigned to one of the intervention conditions based on their region and could not make a choice in the delivery mode of the intervention. This design offers optimal insight into the consequences for response and attrition when using only 1 of the 2 modes for intervention delivery.

**Figure 2:**
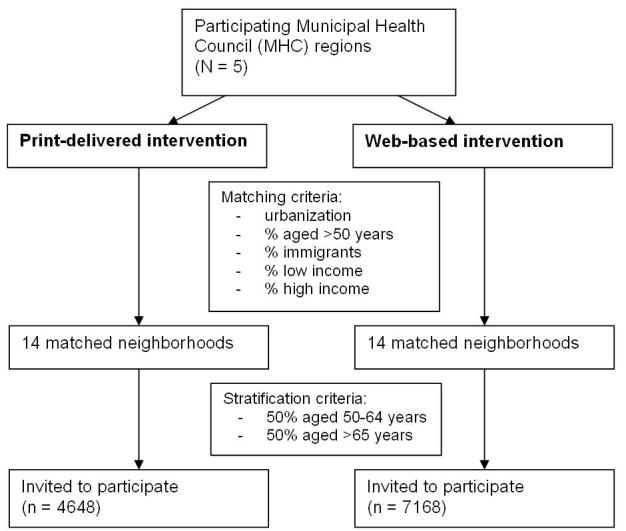
Flowchart showing selection of participants for the print-delivered and Web-based intervention groups.

For each intervention condition, we selected 14 (matched) neighborhoods: 6 less urban neighborhoods (500-1000 addresses per km^2^) and 8 modestly urban neighborhoods (1000-1500 addresses per km^2^, Figure 2). We matched the neighborhoods based on their urban character, percentage of people with a low SES, percentage of people with a high SES, percentage of immigrants (also to ensure that we reached mostly Dutch-speaking people), and the percentage of people over 50 years of age. This information was provided by the Dutch Central Bureau for Statistics. Each MHC provided a random sample of eligible participants living in the selected matched neighbourhoods after stratification for age. Therefore, the distribution of differences in age and SES among the invited adults was expected to be equal between the intervention conditions.

In the regions of the print-delivered intervention, a sample of eligible participants (n=4648) received an invitation for the print-delivered intervention. This invitation included an information letter, a questionnaire, a prepaid return envelope, and a form to give informed consent. Because we expected a lower response in the Web-based intervention conditions, we included a larger sample of eligible participants (n=7168) in the regions of the Web-based intervention. Eligible participants received an invitation via written mail, containing a similar information letter about the project, additional information about how to complete an online questionnaire, and a personal username and password to log on to the Active Plus website. People who did not receive an invitation could not participate in this program. A power calculation (effect size=0.4, power=80%, intracluster correlation coefficient =.1) showed that at baseline about 420 participants were needed for each intervention condition (considering a dropout rate of 40% during the 1-year follow-up based on a previous Active Plus study) [22].

Within both the Web-based and print-delivered intervention groups, participants received the basic tailored intervention or the basic intervention with additional environmentally tailored information, depending on their MHC region. Since the aim of this study was only to investigate how the delivery channel (printed or Web-based) and user characteristics are related to dropout and attrition, and not to determine how the content of the message (eg, providing additional environmental information) influences attrition, both of the print-delivered and both of the Web-based intervention groups were considered together. Dropout analyses were corrected for the possible influence of the intervention type.

Participants were enrolled when they completed the baseline questionnaire. For the second assessment, participants of the print-delivered intervention group received an invitation by written mail, which included the follow-up questionnaire and a prepaid return envelope. Participants of the Web-based intervention group received invitations for the follow-up assessment by email, which included a link to the Web-based questionnaire. All participants were asked to complete the questionnaire within 2 weeks. Participants of the print-delivered intervention group who did not complete the questionnaire received a reminder by mail after 2 weeks. Since the response was lower in the Web-based intervention group than in the print-delivered intervention group and sending reminders electronically does not result in additional postage costs, for the follow-up assessment the Web-based intervention group received multiple reminders (9 days and 18 days after the invitation). Since it was not guaranteed that our target population received the online reminders (eg, redirected to spam folders), this group also received an additional reminder by written mail.

This study is approved by the Medical Ethics Committee of Atrium-Orbis-Zuyd (code 10-N-36) and was registered in the Dutch Trial Register NTR 2297.

### Measurements

The baseline questionnaire included questions about demographics, PA behavior, and sociocognitive determinants of PA behavior. Demographic variables included age, gender, height, weight, and highest education level completed. Education level was categorized into low (primary, basic vocational, or lower general school) and high (higher general secondary education, preparatory academic education, medium vocational school, higher vocational school, or university level), according to the Dutch education system. Body mass index (BMI) was calculated by dividing weight in kilograms by height in metres squared [23]. Participants were classified as being underweight (BMI < 18.5 kg/m^2^), a healthy weight (BMI 18.5–24.9 kg/m^2^), or overweight (BMI > 25 kg/m^2^).

Total weekly days and minutes of PA were measured using the validated self-administered Dutch Short Questionnaire to Assess Health Enhancing Physical Activity (SQUASH) [31]. This questionnaire allows the calculation of the total minutes of moderate- and vigorous-intensity activity per week. It also helps classify participants according to the international guideline for sufficient PA (PA guideline), which is being physically active with moderate to vigorous intensity for at least 30 minutes per day at least 5 days per week [32,33]. The participants’ intention to be sufficiently physically active (according to the international PA guideline) was assessed using three items on a 10-point scale (eg, “To what degree do you intend to be sufficiently physically active?” 1 = Absolutely not, to 10 = Absolutely).

For process evaluation purposes, we asked participants whether they had actually read the advice (“Yes” or “No”) in the second questionnaire (after 3 months, following the first and second sets of tailored advice) and again in the third questionnaire (after 6 months, following the third provision of tailored advice).

### Statistical Analysis

#### Response and Baseline Characteristics of the Study Population

We analyzed data for participants who completed the baseline questionnaire. We performed descriptive statistics on age, gender, education level, BMI, baseline PA, and intention to be sufficiently physically active to describe the characteristics of the participants. We used univariate one-way analyses of variance (ANOVA) and chi-square tests to determine whether the participants in the print-delivered and Web-based interventions differed on baseline characteristics. Furthermore, we used a chi-square test to find out whether the intervention types (ie, the basic tailored intervention or the tailored intervention with additional environmental information) were equally distributed to the print-delivered and Web-based intervention groups. There was no need to correct baseline data for the intervention type because the content of the baseline questionnaire was similar for all intervention groups.

#### Dropout Analysis

We performed hierarchical logistic regression analyses to determine whether participants’ characteristics were predictors of dropout at the 3-month questionnaire, correcting for intervention type (ie, basic or environmental intervention). We added the user characteristics (ie, age, gender, SES, BMI, intention, and PA behavior) to the second block of the regression analysis. To study whether the delivery mode of the intervention was related to participant dropout, we added the intervention delivery mode to the third block of the regression analysis. The influence of the delivery mode on participant dropout was thus corrected for by user characteristics. The fourth block of the regression analysis contained the interaction terms between the delivery mode and the user characteristics, to determine whether participant dropout was related to certain user characteristics in a specific delivery mode. Because the interaction terms have less power, the significance levels the interaction terms are defined at *P*=.10 [34]. When a significant interaction term was found, we performed subgroup analyses separately for the print-delivered and Web-based groups. All analyses were performed using SPSS version 18.0.

## Results

### Response and Baseline Characteristics of the Study Population

**Figure 3:**
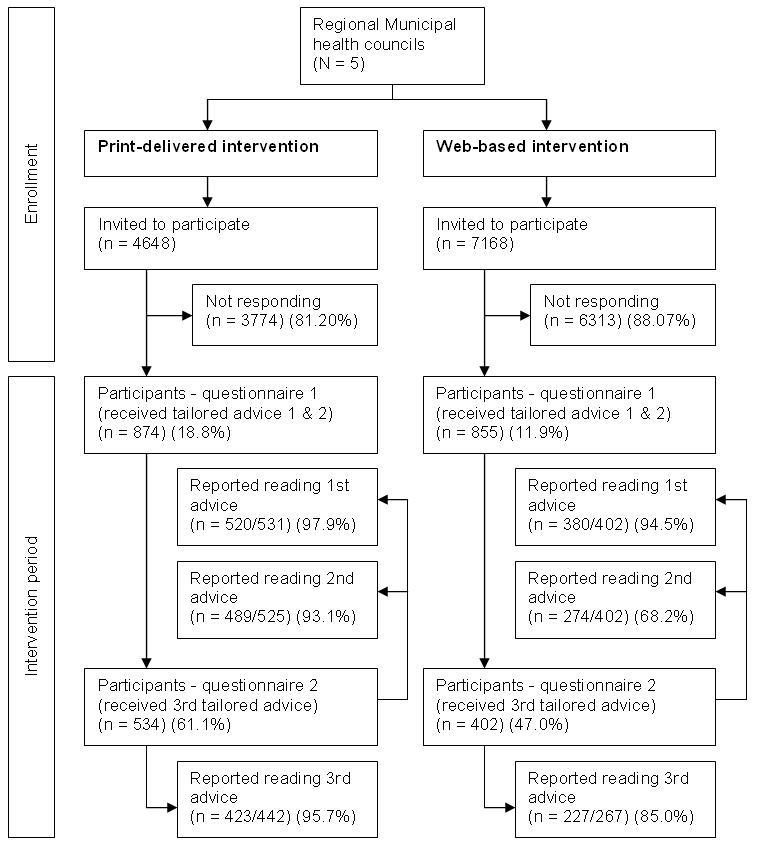
Flow diagram showing the reach, attrition, and usage of the print-delivered and Web-based Active Plus interventions.

A total of 874 adults participated in the print-delivered intervention (response rate of 18.8%) and 855 adults participated in the Web-based intervention (response rate of 11.9%, Figure 3). Baseline characteristics for both intervention groups are shown in Table 1. We found significant differences between the intervention groups with respect to several characteristics. The sample for the Web-based intervention consisted of more men than the sample for the printed intervention (*P*=.01). Participants in the Web-based intervention were significantly younger (*P*<.001) and had a significantly higher BMI (*P*=.001) than the participants of the print-delivered intervention. We found no significant differences between low and high education level among the participants. Regarding PA behavior, the total minutes of moderate to vigorous PA did not differ significantly between the groups. The print-delivered intervention group had a significantly higher intention to be sufficiently physically active (*P*=.03). Participants of the print-delivered and Web-based intervention groups were equally distributed with respect to the basic and the environmental intervention types.

**Table 1:** Sociodemographic and behavioral baseline characteristics for the print-delivered and Web-based intervention groups.

			Print-delivered tailored advice(n=874)	Web-based tailored advice(n=855)	*P* value
**Demographics**					
	Gender (%)				.01
		Male	45.7	51.7	
		Female	54.3	48.3	
	Mean age in years (SD)		63.5 (9.06)	61.3 (7.32)	<.001
	Weight category (%)				.001
		Underweight	1.9	0.4	
		Healthy weight	47.5	42.6	
		Overweight	50.6	57.0	
	Education (%)				.51
		Low	45.4	47.0	
		High	54.6	53.0	
**PA**					
	Moderate- and vigorous-intensity PA, mean minimum/week (SD)		755.83 (786.92)	741.74 (840.65)	.72
	Intention to be sufficiently physically active, mean^a^(SD)		7.73 (1.63)	7.56 (1.58)	.03
**Intervention type (%)**					.81
	Basic intervention		50.1	49.5	
	Environmental intervention		49.9	50.5	

^a^Likert scale, from 1 (“absolutely not”) to 10 (“absolutely”)

### Dropout Analysis

Of the Web-based intervention group, 402 respondents (402/855, 47.0% of baseline participants) completed the second questionnaire and received their third piece of tailored advice. In the print-delivered intervention group, 534 respondents (534/874, 61.1% of baseline participants) completed the second questionnaire. Figure 3 shows an overview of the response and attrition during this study. The figure also provides an insight into the number of participants who, in addition to filling in the second questionnaire, reported that they actually read the tailored advice (based on process evaluation data retrieved from the second and third questionnaires). The percentage of participants who reported to have read the advice is expressed as the number of people who reported to have read the advice, divided by the number of people who filled in the program evaluation question. The number of participants who reported that they read their advice was substantially smaller in the Web-based intervention than in the print-delivered intervention (*P*<.001).

At the 3-month measurement, significant differences in user characteristics between both intervention groups remained the same as the baseline measurement. Table 2 provides an overview of the predictors of participant dropout within the intervention period (ie, filling in the 3-month questionnaire). The explained variance (Nagelkerke *R* ^*2*^) of the different models is also presented in this table. The participants’ intention to be physically active and the delivery mode of the intervention were both significant predictors of participant dropout. A higher intention was positively related to the completion of the intervention (*P*<.001), and participants of the Web-based intervention were more likely to drop out (*P*<.001). No other demographic characteristics (ie, baseline PA, age, SES, gender, BMI, and intervention type) were predictors of participant dropout. We found a significant interaction between age and the delivery mode (Exp(B)=1.026, SE=0.013, *P*=.05). No other significant interaction terms were found between the delivery mode of the intervention and the participants’ characteristics. Subgroup analyses showed that in the print-delivered intervention group, age was not a significant predictor of participant dropout (Exp(B)=0.993, SE=0.009, *P*=.45). Analyses for the Web-based intervention group showed that a higher age might be an indicator for completion of the intervention (Exp(B)=1.019, SE=0.010, *P*=.05).

**Table 2:** Hierarchical logistic regression to study the relation between user characteristics, the intervention delivery mode and its interactions, in the prediction of attrition within the intervention period.^a^

		Step 1 (*R* ^*2*^=.001)			Step 2 (*R* ^*2*^=0.024)			Step 3 (*R* ^*2*^=0.051)			Step 4 (*R* ^*2*^=0.058)		
		Exp(B)	SE	*P*	Exp(B)	SE	*P*	Exp(B)	SE	*P*	Exp(B)	SE	*P*
**First block**													
	Type^b^	0.876	0.099	.18	0.881	0.100	.21	0.884	0.101	.22			
**Second block**													
	Baseline PA				1.000	0.000	.45	1.000	0.000	.45			
	Intention				1.171	0.033	.000	1.159	0.033	.000			
	Age				1.010	0.006	.12	1.005	0.006	.48			
	SES^c^				0.956	0.104	.66	0.934	0.105	.51			
	Gender^d^				1.044	0.104	.68	1.007	0.105	.95			
	BMI				0.904	0.100	.31	0.928	0.101	.46			
**Third block**													
	Delivery mode^e^							0.550	0.102	.000			
**Fourth block**													
	Delivery x PA										1.000	.000	.50
	Delivery x Intention										.951	.067	.45
	Delivery x Age										1.026	.013	.05
	Delivery x SES										.985	.212	.94
	Delivery x Gender										.855	.211	.46
	Delivery x BMI										1.304	.203	.19

^a^participants scoring 1 are more likely to complete the intervention, whereas scores of 0 indicate that participants are more likely to dropout

^b^basic coded 0, environmental coded 1

^c^low SES coded 0, high SES coded 1

^d^men coded 0, women coded 1

^e^printed coded 0, Web-based coded 1

## Discussion

### Response

The present study aimed to assess differences in user characteristics related to participation and attrition when comparing a print-delivered intervention and Web-based intervention to stimulate PA among people over 50 years of age. Our study showed that, in this population, the response rate to the print-delivered PA intervention (n=874/4648, 18.8%) was higher than the response rate to the Web-based intervention (n=855/7168, 11.9%). This finding indicates that using a computer might still be a barrier to participation. The difference in response rate may be due to a lack of motivation or limited skills in using the Internet among the target population. This difference is also acknowledged by Venkatesh’s unified theory of acceptance and use of technology (UTAUT). According to this theory, performance and effort expectancy explain a large proportion of the variance in the intention to use a new technology [35]. This may imply that older adults’ skills and self-efficacy in computer usage need to be increased to stimulate the adoption of Web-based interventions. Future researchers should study these barriers further, and may need to incorporate additional information or computer training in their recruitment or intervention to increase the computer skills and self-efficacy of older adults. However, differences in self-efficacy and computer skills among generations might decrease rapidly, since the adults of the current generation have more developed computer skills and are the elderly of the future.

Response rates in Web-based interventions reported in the literature vary substantially between studies. Differences can be caused either by the methodology of the study or by the characteristics of the population. Researchers have conducted several Web-based interventions in populations with known access to the Internet, which resulted in higher response rates than studies in populations without known access [15]. Discovering a lower response rate to a Web-based intervention compared to a print-delivered intervention is in agreement with the findings of a study by Kongved et al [15] that was conducted in a population without known Internet access. The study further showed that after a reminder, when the participants were free to choose between delivery modes, the total response rate was similar in the 2 groups. Similarly, a study by Ekman et al [36] showed that response rates were highest when 2 response methods (ie, print-delivered and Web-based) were offered. In our study, participants of the Active Plus intervention could not choose between delivery modes because different intervention conditions were located in different regions, which prevented participants contaminating each other’s responses. Offering both delivery modes to the participants might have resulted in higher response rates, but would not have provided insight into the actual use of different single-delivery modes.

Another reason for the low response rate could be the length of our questionnaire. A review [37] to identify effective strategies to increase the response to postal and electronic questionnaires showed that the odds of response increased by more than half when participants received shorter questionnaires. The response rate improved with shorter print-delivered questionnaires (odds ratio (OR) 1.64) as well as in studies using electronic questionnaires (OR 1.73), compared to participants given longer questionnaires. Due to research requirements, filling in the Active Plus questionnaires took quite some time (the baseline questionnaire is about 24 pages). In a real-life setting, when no additional questions for research purposes are required, the length of the questionnaires can be shortened, which might result in a higher response rate and limit the dropout rates of the intervention.

We found significant differences between the print-delivered and Web-based intervention groups with respect to several characteristics. The sample for the Web-based intervention was significantly younger and consisted of more men. Both findings can be supported by epidemiological evidence that shows that Internet access is still lower in the over 65 age group, and that men use the Internet more frequently and intensively than women [7,8].

Furthermore, participants of the Web-based intervention had a significantly higher BMI and a lower intention to be sufficiently physically active. This might indicate that adults over 50 years of age who have a low intention to be sufficiently physically active might best be reached using a Web-based intervention. Together with the finding that the majority (480/842, 57.0%) of the Web-based intervention group had an unhealthy BMI, Web-based interventions might be the preferred medium to reach this high-risk population. These differences in user characteristics between print-delivered and Web-based intervention groups should also be taken into consideration when determining the possible effects of the intervention in different delivery modes. However, differences in participant dropout rate between both delivery modes should also be acknowledged.

In contrast to our expectations, no differences were found in education level between the Web-based and print-delivered intervention groups. This is in contrast to several other studies that show that Web-based interventions have a higher reach among high SES populations [38,39]. Possibly, education level is less important to Internet usage in an older population than in the general population. This finding can be explained further by the fact that one of the highest shares of home Internet access in Europe was recorded in the Netherlands (94% in 2011) [8], where differences in Internet access between SES groups has declined rapidly. As epidemiological data shows, in 2010, at the moment of implementing the Active Plus interventions, 87% of the low SES group and 98% of the high SES group in the general population had Internet access [7]. In 2011, the low SES group further increased their Internet access to 90% [7].

### Dropout

Since the Active Plus intervention contains multiple provisions of advice, it is important that people continue their participation in the intervention. Our results show that only 61.1% (534/874) of the print-delivered intervention group and only 47.0% (402/855) of the Web-based intervention group filled in the second questionnaire and were thus eligible to receive the third follow-up tailored advice. Furthermore, the number of participants who reported reading their advice was substantially smaller in the Web-based intervention than in the print-delivered intervention. Participant dropout within the intervention period was therefore significantly higher in the Web-based intervention group than in the print-delivered intervention group. The difference in participant dropout rate between the intervention conditions was not explained by user characteristics. We found a significant interaction between the delivery mode and the participants’ age in the prediction of participant dropout. Subgroup analyses showed that whereas age was not a predictor of participant dropout in the print-delivered group, there was an indication (almost significant predicator (*P*=.05)) that younger participants were more likely to drop out from the Web-based intervention. The finding that older adults are more likely to revisit the website is in line with other studies [12,40].

In both delivery modes, a low intention to be physically active was a significant predictor of participant dropout. It is self-evident that people with a lower intention to remain physically active are less likely to continue their participation. Since we also found that low intention participants more often participate in Web-based interventions, this would consequently lead to a higher dropout rate for Web-based interventions. Hence, additional strategies are needed to motivate adults with a low intention to continue their participation in health-promoting interventions. If these participants are less motivated by their health intention, this group needs additional motivators to continue. For example, motivation could be improved by making the intervention more exciting by including gaming elements or by focusing on other aspects besides health, such as news elements, sports games, or social activities.

Since participant dropout was significantly higher in the Web-based intervention and no significant factor was found for user characteristics explaining the differences in dropout rate between the delivery modes, the higher dropout in Web-based interventions must be related to other characteristics. A possible explanation is that it might require more planning to fill in a Web-based questionnaire than a print-delivered questionnaire. A print-delivered questionnaire can be filled in anywhere at anytime, while filling in an online questionnaire restricts one to a computer. Furthermore, in a printed questionnaire, the time required to fill in the questionnaire is more visible (you see the total package in one overview), and it is easier to continue filling in a printed questionnaire after pausing, rather than continuing a Web-based questionnaire, due to loading times and additional log-ins.

### Strengths and Limitations

Although this study provides interesting data, some limitations should be noted. First of all, no information was available on those who did not respond to the intervention. We could have performed more predictive analyses if information about the nonparticipants was available to provide insight into the selection process. Second, participants could not choose between the different modes of delivery. They could only choose whether they would like to participate in the intervention with the particular delivery mode that was offered. Giving participants the option to choose between the delivery modes could provide additional insights into the reasons why people participate in a certain intervention. However, not allowing participants to choose between the delivery modes is also a strength—it gave us the opportunity to study the resultant absolute participation and attrition rates for a given delivery mode. In a real-life setting, an intervention is usually offered in only 1 delivery mode because offering both delivery modes results in additional planning and administration. The current design offers optimal insight into the consequences of using 1 of the 2 delivery modes.

Another limitation of this study is that the program evaluation (to assess whether the participants had read the advice) took place several months after the participants received their tailored advice. As a result, some recall bias might have occurred. For future evaluation studies, we recommend sending a short program evaluation questionnaire after each tailored advice to limit recall bias, or to include multiple questions to validate their report.

To our knowledge, this is the only study that compares the characteristics of users related to the use and participant dropout rate of a Web-based intervention and a print-delivered intervention with similar content in an older population. Since older adults are one of the fastest growing online user groups and tend to have the most interest in and need for health advice compared to other age groups [9,10], it is important to gain more insight into this population’s user characteristics. Furthermore, we conducted our study on a large and diverse target population in which both low and high SES subgroups were represented. By stratifying invitees by age and SES at a neighborhood level, we could compare the response rates of the print-delivered and Web-based intervention conditions.

### Conclusion

The results of our study suggest that the response to a Web-based intervention is significantly lower than a print-delivered intervention among Dutch people over 50 years of age, and participants with different characteristics were attracted by different delivery modes. Participants with a low intention to be physically active and a high BMI were more attracted by the Web-based intervention, indicating that Web-based interventions might be a good medium to reach this high-risk population.

Although participant dropout was significantly higher in the Web-based intervention, no significant factors were found for user characteristics explaining the differences in participant dropout between the delivery modes. This indicates that the higher dropout rate in Web-based interventions is potentially relevant for all demographic groups and related to characteristics not measured in this study.

Our study has provided important new insights into the differences in user characteristics of participants in a print-delivered or Web-based intervention in an older population. These findings are important when selecting the delivery mode of an intervention that aims to optimize exposure to a certain subgroup and when interpreting and generalizing results of randomized controlled effectiveness trials. Since Internet use is growing rapidly in older populations, more research is needed to explore the prolonged use, appreciation, and effectiveness of Web-based interventions compared to print-delivered interventions in this population. The low cost of Web-based interventions can provide an opportunity to reach more of the population, if the effectiveness of the intervention method can be improved. Furthermore, more research is needed to investigate strategies that limit the high dropout rates in Web-based interventions (especially among participants with a low PA intention) and to ensure a sustained intervention effect.

## Abbreviations

ANOVA: univariate one-way analyses of variance
BMI: body mass index
MHC: Municipal Health Council
OR: odds ratio
PA: physical activity
RCT: randomized controlled trial
SES: socioeconomic status
SQUASH: short questionnaire to assess health enhancing physical activity
UTAUT: Venkatesh’s unified theory of acceptance and use of technology
